# Influence Factors and Prediction Model of Bond Strength of Tunnel Fireproof Coating under Freeze–Thaw Cycles

**DOI:** 10.3390/ma14061544

**Published:** 2021-03-22

**Authors:** Guanji Lu, Tao Ji, Binbin Zhang, Yu Ma, Cong Liao

**Affiliations:** 1 Department of Management Engineering, Fujian Business University, Fuzhou 350012, China; 2 College of Civil Engineering, Fuzhou University, Fuzhou 350108, China; jt72@fzu.edu.cn (T.J.); ZBB102766@163.com (B.Z.); benxiaohao2021@163.com (Y.M.); Liaoc2003@163.com (C.L.)

**Keywords:** tunnel fireproof coating, bond strength, prediction model, pore structure, freeze–thaw cycles

## Abstract

The freeze–thaw resistant performance of a tunnel fireproof coating (TFC) has an important impact on bonding property and durability. The influence of redispersible emulsion powder, polypropylene fiber and air-entraining agent on TFCs was studied. Transverse fundamental frequency and ultrasonic sound velocity were used to evaluate the damage degree of TFC, and the mechanism was revealed by SEM and pore structure. The results show that the most beneficial effect on bond strength of TFC is redispersible emulsion powder, followed by air-entraining agent, and then polypropylene fiber. After freeze–thaw cycles, the cumulative pore volume of micropores in the TFC increases obviously, while the porosity of macropores does not change significantly. A prediction model was proposed, which can calculate the bond strength from the damage degree of TFC under freeze–thaw cycles. The achievement can promote the application of TFC in cold regions.

## 1. Introduction

At present, the main measure for tunnel structure fire protection uses a fireproof coating (TFC) as the tunnel structure fire-resistance layer. Research on the cohesiveness, water-resistance and fire-resistance of TFCs has made considerable progress. However, the problem of bonding performance of TFCs in tunnels under freeze–thaw cycles is raised together with the problem of disease in tunnels. When a TFC suffers freeze damage, the TFC expands and then begins to loosen, piles up, or even peels off under repeated freeze–thaw action. This not only makes the TFC not only unable to play a fire insulation function but also becomes a tunnel safety hazard. Therefore, it is necessary to study the relationship between bonding performance and damage of TFC under freeze–thaw cycles.

The fireproof tunnel coating is a coating product specifically for tunnel fireproofing, along with the development history of the fireproof coating. The development of fireproof coatings began with the first fireproof coatings developed by Louis Pacmboenf in 1837 [1]. With the growth of time, several studies on the performance of tunnel fireproof coatings were found in the literature [2,3,4,5,6,7,8,9]. Weil. E et al. [10] developed new inorganic building fireproof coatings based on cement, inorganic mineral fiber, expanded perlite, expanded vermiculite and light calcium carbonate. Kidder et al. [11] developed fireproof coatings with excellent thermal insulation and explosion-proof performance for high alumina cement, fiber, sepiolite, calcium silicate and amorphous silica for tunnel structures and building exterior walls. The previous studies only focus on another durability of fireproof coatings, while the study of freezing damage to tunnel fireproof coatings is relatively rare. Meanwhile, the Chinese standard [12] stipulates that the bond strength of the coating shall be not less than 0.15 MPa after 15 freeze–thaw cycles.

At present, there are few studies on freeze–thaw damage of tunnel fireproof coatings, and no literature on freeze–thaw damage of tunnel fireproof coatings has been found. Many scholars [13,14,15,16,17,18,19,20,21,22,23,24,25,26,27,28] have established an elastoplastic damage model for predicting frost resistance of concrete by introducing the plastic increment theory of stiffness, strength degradation and residual deformation. Cai Hao [29] proposed a theoretical damage model on the basis of Loland’s unidirectional tensile damage evolution model; on the basis of regression analysis of material parameters, some empirical models were obtained, such as the concrete durability index relationship established by Xu Liping [30] considered the air content. In short, there is no relevant research on the prediction model between bond strength and damage degree of TFC. In fact, if the freeze–thaw damage law of TFC is clear, the development law of TFC performance (such as bond strength) can be analyzed, and the corresponding prediction formula of frost resistance performance can be obtained, which has a guiding role for the practical engineering application of TFC.

Some scholars [31,32,33] have studied the bond properties of TFC without freeze–thaw cycles, but there is little research on the bond performance after freeze–thaw cycles. The paper investigates the effects of redispersible emulsion powder, polypropylene fiber and air-entraining agent on the performance of TFC under freeze–thaw cycles. Transverse fundamental frequency and ultrasonic sound velocity were used to evaluate the damage degree of TFC. ESEM was used to analyze the changes of macro and micropore structure in TFC, and the mechanism of bond performance under freeze–thaw cycle was explained. Meanwhile, the model between bond performance and damage degree is established. The achievement of this paper can promote the application of TFC in cold regions.

## 2. Experimental

### 2.1. Materials

The fineness of polyvinyl alcohol (PVA) is 0.090–0.110 mm, which is a white powder, odorless and tasteless. The air-entraining agent is a white powder used to improve the freeze–thaw resistance of coatings. It is made in Basel, Switzerland, and the best content is 0.01–0.03%. The technical parameters of cement, high-alumina cement, redispersible emulsion powder and polypropylene fiber are shown in Table 1, Table 2, Table 3, Table 4 and Table 5, and their source information can be found in this article [34]. The technical parameters and source information of other material, including polyvinyl alcohol, polyvinyl alcohol silyl powder, expanded vermiculite, expanded perlite, sepiolite, hollow floating bead, Mg(OH)_2_, Al(OH)_2_, melamine, pentaerythritol, can be found in the paper [34].

### 2.2. Test Mix Proportion

The content of redispersible emulsion powder ($\alpha_{1}$) was 0%, 2% and 4%, respectively. The polypropylene fiber content ($\alpha_{2}$) was 0%, 0.2% and 0.4%, respectively. The content of air-entraining agent ($\alpha_{3}$) was 0%, 0.015% and 0.030%, respectively. The parameter level and test mix proportion are shown in Table 6 and Table 7, respectively. The dry material (M) is the sum of the mass of M1–M15 in Table 6. The sum of M1–M15 is 100%, and the air-entraining agent was mixed externally; others were mixed internally.

(1) $$\alpha_{1}{=m}_{1}/M$$

(2) $$\alpha_{2}{=m}_{2}/M$$

(3) $$\alpha_{3}{=m}_{3}/M$$

m_1_, m_2_ and m_3_ are the mass of redispersible emulsion powder, polypropylene fiber and air-entraining agent, respectively.

### 2.3. Specimen Preparation and Test Methods

#### 2.3.1. Freeze–Thaw Cycles Test

There were three kinds of specimens subjected to the freeze–thaw cycle, namely, plate specimens, bond strength specimens and cube specimens. The size of the plate specimen was 150 mm × 70 mm × 6 mm, the thickness of the TFC was (5 ± 1) mm. Three of each sample were used to observe whether the coating was cracked, peeled or discolored during the freeze–thaw cycles. The bond strength specimen included the base plate and TFC. The base plate size of the bond strength specimen was 70 mm × 70 mm × 6 mm, the TFC thickness was (5 ± 1) mm, and the number of each specimen was 3. The cube specimen was 100 mm × 100 mm × 100 mm, and the number of each sample was 3. It was used to measure the mass of TFC, transverse fundamental frequency and ultrasonic velocity under freeze–thaw cycle. The preparation process of specimens met the relevant Chinese standards [35].

#### 2.3.2. Mass Loss Rate Test

At the end of the freeze–thaw cycles, we took out the specimen and dried the surface. We measured the mass and calculated the mass-loss rate according to the standard formula [12]. We calculated the mass-loss rate as the ratio of mass-loss to initial mass after freeze–thaw cycles.

#### 2.3.3. Damage Degree Test

A DT-20 dynamic elastic instrument (made in Gangyuan test instrument factory, Tianjin, China) was used to measure the transverse fundamental frequency of TFC according to the standard of China [35]. An NM-4A nonmetal ultrasonic testing analyzer (made in Kangkerui company, Beijing, China) was used to obtain ultrasonic velocity.

#### 2.3.4. Bond Strength Test

The test instrument was made in Beijing, China. The test method was in accordance with the requirements of Chinese standards [36].

#### 2.3.5. Test Method of Pore Structure

The micropore structure was determined by the nitrogen adsorption method. F-sorb2800 pore structure analyzer was used in the test, which was made in Kingalp company, Beijing, China. The test sample was crushed into particles with a diameter of about 3 mm. After weighing with an electronic balance, we took about 3 g of sample particles and put them into the sample tube. The macropore structure was processed by Matlab image processing technology, and the sample was sliced from the TFC before and after freeze–thaw cycle. The camera used in the experiment was Nikon d7000 SLR.

#### 2.3.6. Environmental Scanning Electron Microscope Test (ESEM)

The samples were made into 10 mm × 10 mm × 10 mm according to the mix proportion of the D4 sample in Table 7, and the ESEM test was carried out after curing for 28d. The type of environmental scanning electron microscope is XL30, which was produced in FEI company, Holland.

## 3. Results and Discussion

### 3.1. Bond Strength

**Figure 1:**
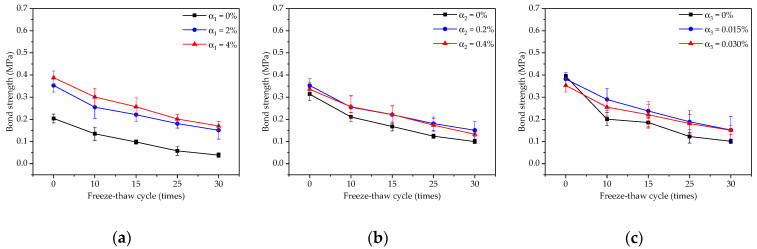
Influence of three factors on bond strength: (**a**) the factor of redispersible emulsion powder; (**b**) the factor of polypropylene fiber; (**c**) the factor of air-entraining agent.

The variation rules of bond strength of coatings under freeze–thaw cycles are shown in Figure 1a by comparing the samples of D1, D4 and D5. Only the content of redispersible emulsion powder changed; the other two factors remained unchanged in Figure 1a. When the content of redispersible emulsion powder increased from 0% to 2% and 4%, the initial bond strength of coatings increased by 73.0% and 90.2%, respectively, indicating that the redispersible emulsion powder was beneficial to improve the initial bond strength of coatings. When the freeze–thaw cycles were up to 15 times, the bond strength with 0%, 2% and 4% redispersed emulsion powder was 0.098 MPa, 0.221 MPa and 0.257 MPa, which decreases by 52.0%, 37.4% and 33.8% compared with the initial bond strength, respectively. When freeze–thaw cycles were up to 30 times, the bond strength with 0%, 2% and 4% redispersible emulsion powder was 0.039 MPa, 0.151 MPa and 0.170 MPa, which decreased by 81.2%, 57.1% and 56.0% compared with the initial bond strength, respectively. This indicated that the addition of redispersible emulsion powder could improve not only the initial bonding strength of the coating but also benefitted the frost-resistance of the TFC.

The variation rules of bond strength of coatings under freeze–thaw cycles are shown in Figure 1b by comparing the samples of D2, D4 and D6. Only the content of polypropylene fiber changed; the other two factors remained unchanged in Figure 1b. The initial bond strength of TFC with the polypropylene fiber content of 0%, 0.2% and 0.4% were 0.314 MPa, 0.353 MPa and 0.336 MPa, respectively. When the content of polypropylene fiber was from 0% to 2% and 4%, the initial bond strength increased by 12.4% and 7.0%, respectively. With the increase of freeze–thaw cycle, the bonding strength of TFC decreased. When the freeze–thaw cycles reached 30 times, the bond strength of TFC with 0%, 0.2% and 0.4% polypropylene fibers was 0.100 MPa, 0.151 MPa and 0.133 MPa, respectively, which decreased by 68.1%, 57.1% and 60.6% compared with the corresponding initial bond strength, respectively.

The variation rules of bond strength of coatings under freeze–thaw cycles are shown in Figure 1c by comparing the samples of D3, D4 and D7. Only the content of the air-entraining agent changed; the other two factors remained unchanged in Figure 1c. The initial bond strength of the coating was 0.394 MPa, 0.382 MPa and 0.353 MPa, with a slight decrease when the content of the air-entraining agent increased from 0% to 0.015% and 0.030%. With the increase of freeze–thaw cycles, the bonding strength of TFC decreased. When the freeze–thaw cycles were up to 15 times, the bond strength of TFC under the three concentrations of 0%, 0.015% and 0.030% of the air-entraining agent was 0.201 MPa, 0.290 MPa and 0.255 MPa, respectively, which decreased by 52.9%, 37.6% and 37.3%, compared with the corresponding initial bond strength. When freeze–thaw cycles reached 30 times, the bond strength of coatings with 0%, 0.015% and 0.030% dosage was 0.101 MPa, 0.152 MPa and 0.151 MPa, which decreased by 74.3%, 60.2% and 57.1%, compared with the corresponding initial bond strength.

### 3.2. Mass Loss Rate

**Figure 2:**
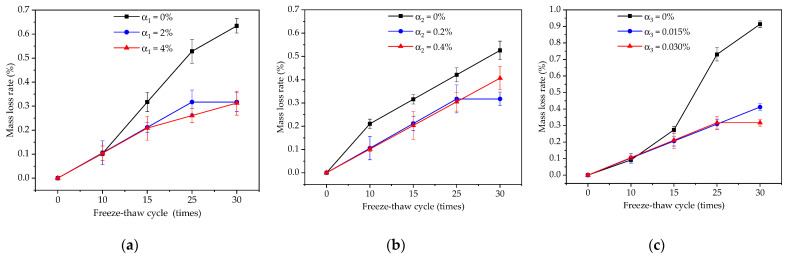
Influence of three factors on the mass-loss rate: (**a**) the factor of redispersible emulsion powder; (**b**) the factor of polypropylene fiber; (**c**) the factor of air-entraining agent.

The variation rules of mass-loss rate of the coating under freeze–thaw cycles are shown in Figure 2a by comparing the samples of D1, D4 and D5. Only the content of redispersible emulsion powder changed; the other two factors remained unchanged in Figure 2a. When the freeze–thaw cycle was less than 10 times, the mass-loss rate of the coatings with 0%, 2% and 4% redispersible emulsion powder was almost the same. However, when the freeze–thaw cycles were more than 10 times, the mass-loss rate of the coating with 0% content was the largest, and the mass-loss rate of the coating with 2% and 4% content was almost the same, which was different only when the freeze–thaw cycles were about 25 times. The results show that the mass-loss rate of 2% and 4% redispersible emulsion was 10.6% and 31.7%, respectively, compared with that of 0% in 15 and 30 freeze–thaw cycles. With the increase of freeze–thaw cycles, the mass-loss rate of the coating increased, but the mass-loss rate of 2% and 4% redispersible emulsion powder was obviously slower than that of 0%.

The variation rules of mass-loss rate of coatings under freeze–thaw cycles are shown in Figure 2a by comparing the samples of D2, D4 and D6. Only the content of polypropylene fiber changed; the other two factors remained unchanged in Figure 2b. When the freeze–thaw cycle was 15 and 30 times, the mass-loss rate of the coating with a polypropylene fiber content of 0.2% was 10.4% and 20.9% less than that with 0%, respectively. The mass-loss rate of the coating with a polypropylene fiber content of 0.2% and 0.4% was almost the same, only when the freeze–thaw cycle was 30 times. With the increase of freeze–thaw cycles, the mass-loss rate of the coating increased.

The variation rules of mass-loss rate of coatings under freeze–thaw cycles are shown in Figure 2c by comparing the samples of D3, D4 and D7. Only the content of the air-entraining agent changed; the other two factors remained unchanged in Figure 2c. When the freeze–thaw cycle was less than 10 times, the mass-loss rate of the coatings with 0%, 0.015% and 0.030% air-entraining agent was almost the same. However, when the freeze–thaw cycles were more than 10 times, the mass-loss rate of the coating with 0% content was the largest, and the mass-loss rate of the coating with 0.015% and 0.030% content was almost the same, which was different only when the freeze–thaw cycles reached 30 times. With the increase of freeze–thaw cycles, the mass-loss rate of the coating increased, but the mass-loss rate of 0.015% and 0.030% air-entraining agent was obviously slower than that of 0%.

### 3.3. Damage Degree

**Figure 3:**
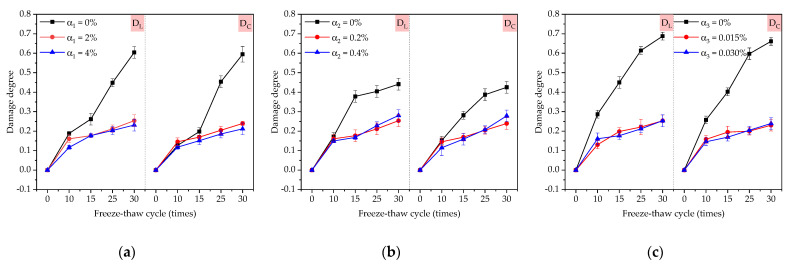
Influence of three factors on damage degree: (**a**) the factor of redispersible emulsion powder; (**b**) the factor of polypropylene fiber; (**c**) the factor of air-entraining agent. D_L_: damage degree measured by transverse fundamental frequency; D_C_: damage degree measured by supersonic velocity.

The variation rules of damage degree of coatings under freeze–thaw cycles are shown in Figure 3a by comparing the samples of D1, D4 and D5. Only the content of redispersible emulsion powder changed; the other two factors remained unchanged in Figure 2a. The changed rule of D_L_ and Dc was basically the same, and it increased with the increase of freeze–thaw cycles. At 15 and 30 freeze–thaw cycles, the damage degree of 2% redispersible latex powder was 0.029 and 0.36 less than that of 0%, respectively. When the freeze–thaw cycle was 30 times, the damage degree of TFC with redispersible emulsion powder content of 0% was about three times compared with that of 4%. The damage degree of 2% and 4% redispersible emulsion powder was almost the same.

The variation rules of damage degree of coatings under freeze–thaw cycles are shown in Figure 2b by comparing the samples of D2, D4 and D6. Only the content of polypropylene fiber changed; the other two factors remained unchanged in Figure 2b. Polypropylene fiber could reduce the damage of TFC in freeze–thaw cycles, but when the content of polypropylene fiber was greater than 0.2%, the damage reduction effect was not obvious. When the freeze–thaw cycles were less than 10 times, the damage degree of the coatings with a polypropylene fiber content of 0%, 0.2% and 0.4% were close. When the freeze–thaw cycles were more than 10 times, the damage degree of the coating was greatly reduced when the polypropylene fiber content was 0.2%.

The variation rules of damage degree of coatings under freeze–thaw cycles are shown in Figure 3c by comparing the samples of D3, D4 and D7. Only the content of the air-entraining agent changed; the other two factors remained unchanged in Figure 3c. The changed rule of D_L_ and Dc was basically the same. At 0, 15, and 30 freeze–thaw cycles, the damage degree of 0.015% air-entraining agent was 0.21 and 0.43 less than that of 0%, respectively. Unlike redispersible emulsion powder and polypropylene fiber, the damage reduction effect of the air-entraining agent was already evident at the beginning of the freeze–thaw cycles.

### 3.4. Microscopic Pores

**Figure 4:**
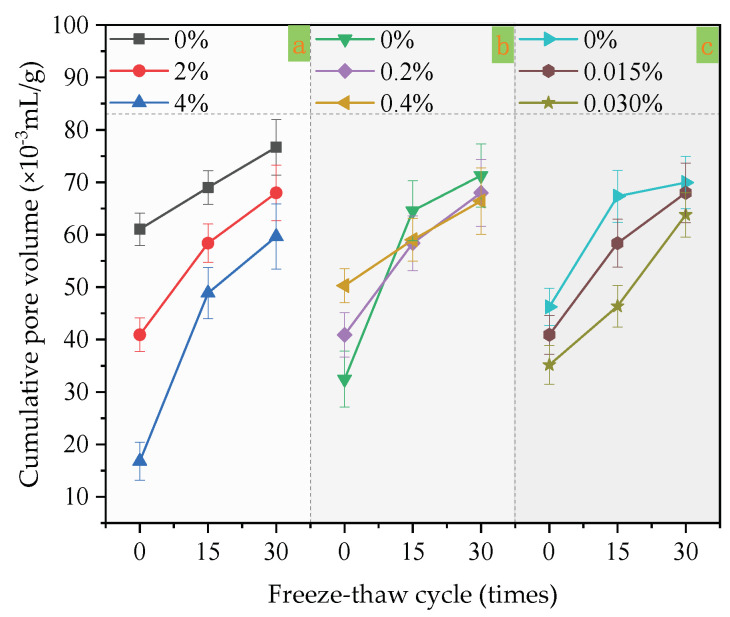
Influence of three factors on cumulative pore volume: (**a**) the factor of redispersible emulsion powder; (**b**) the factor of polypropylene fiber; (**c**) the factor of air-entraining agent.

**Figure 5:**
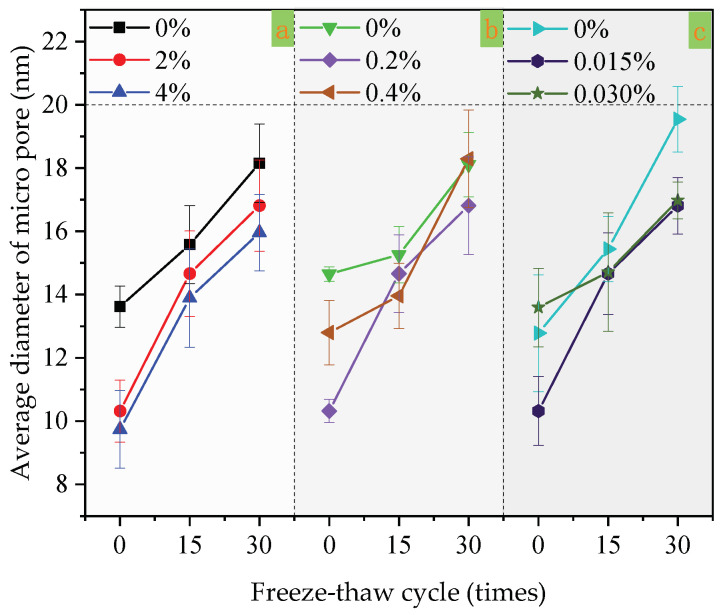
Influence of three factors on the average diameter of micropore: (**a**) the factor of redispersible emulsion powder; (**b**) the factor of polypropylene fiber; (**c**) the factor of air-entraining agent.

In Figure 4a, only the content of redispersible emulsion powder changed, while the content of other factors remained unchanged. Others are also single factor changes in Figure 4b,c and Figure 5a–c. When the content of redispersible emulsion powder increases from 0%, 2% to 4%, the cumulative pore volume and average diameter decrease under the same freeze–thaw cycles (as shown in Figure 4a and Figure 5a). The redispersible emulsion powder is beneficial to improve the freeze–thaw resistance of coatings. Under the same freeze–thaw cycles, the larger the content of redispersible emulsion powder, the smaller the cumulative pore volume and average diameter of micropore.

At 0 freeze–thaw cycles, the cumulative pore volume decreased with the increase of polypropylene fiber content. However, in 15 and 30 freeze–thaw cycles, the cumulative pore volume of the coatings with 0.4% polypropylene fiber content was larger than that with 0%, and the cumulative pore volume of the coatings with 0.2% was basically the same as that with 0%. The average diameter of micropore increased with the increase of freeze–thaw cycles. The average pore diameter of the coating with a polypropylene fiber content of 0.2% was the smallest at 0 and 30 freeze–thaw cycles. Moreover, the coating with 0.4% polypropylene fiber had the smallest average pore diameter in 15 freeze–thaw cycles. Moreover, when the content of polypropylene fiber was 0.2%, the mass-loss rate and damage degree were reduced compared with that 0%.

When the content of the air-entraining agent increased from 0%, 2%, to 4%, the cumulative pore volume decreased under the same freeze–thaw cycles. With the increase of freeze–thaw cycles, the cumulative pore volume and average diameter of the coating increase. The coating with 0.015% polypropylene fiber had the smallest average micropore diameter at 0 freeze–thaw cycles, but the coating with 0.015% polypropylene fiber had the same average micropore diameter as that with 0.030% at 15–30 freeze–thaw cycles.

### 3.5. Macro Pores

**Figure 6:**
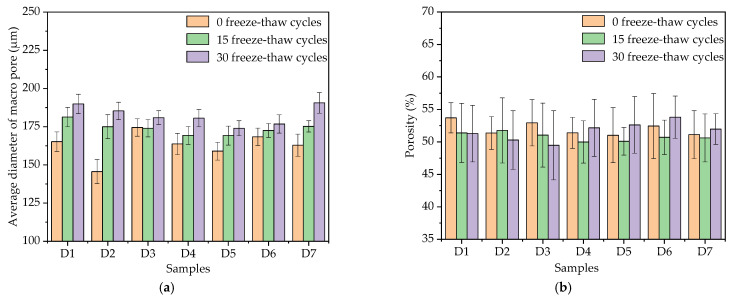
Average macroscopic pore diameter and porosity under freeze–thaw cycles: (**a**) average macroscopic pore diameter under freeze–thaw cycles; (**b**) porosity under freeze–thaw cycles.

**Figure 7:**
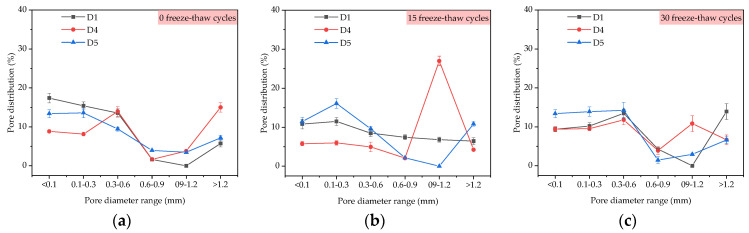
Pore distribution under the influence of redispersible emulsion powder: (**a**) 0 freeze-thaw cycles; (**b**) 15 freeze-thaw cycles; (**c**) 30 freeze-thaw cycles.

From Figure 6a, the average diameters of D1, D4 and D5 were 165.2 μm, 163.7 μm, and 159.0 μm under 0 freeze–thaw cycles, respectively, and the average diameters of D1, D4 and D5 were 181.3 μm, 169.3 μm, and 169.2 μm under 15 freeze–thaw cycles, respectively. The average diameters of D1, D4 and D5, increased by 9.75%, 3.42% and 6.41%, respectively, when the freeze–thaw cycles increased from 0 to 15 times. The average diameters of macroscopic pores in TFC were 189.9 μm, 180.6 μm, 174.0 μm, and increased by 14.95%, 10.32% and 9.43% in 30 freeze–thaw cycles compared with 0 freeze–thaw cycles, respectively. The results show that the addition of redispersible emulsion powder improved the freeze–thaw resistance of macropores. From Figure 6b, at 0 and 15 freeze–thaw cycles, the porosity of the coating with 4% redispersible emulsion powder was the smallest, but at 30 freeze–thaw cycles, the porosity was the largest. From Figure 7, the proportion of large pore size increased slightly with the increase of freeze–thaw cycles. At 15 freeze–thaw cycles, the pore distribution of the D4 sample was 26.98% in the pore diameter range of 0.9–1.2 mm.

**Figure 8:**
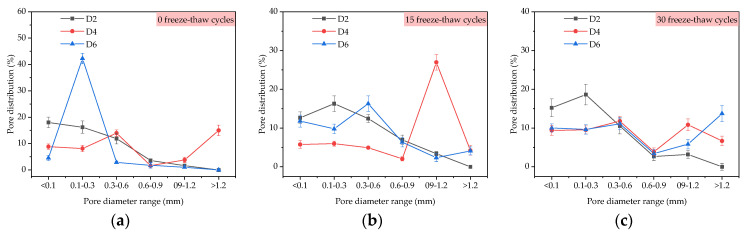
Pore distribution under the influence of polypropylene fiber: (**a**) 0 freeze-thaw cycles; (**b**) 15 freeze-thaw cycles; (**c**) 30 freeze-thaw cycles.

By comparing D2, D4, and D6 samples in Figure 6a, when the polypropylene fiber content in TFC increased from 0%, 0.2% to 0.4% at 15 freeze–thaw cycles, the average diameters of the corresponding macroscopic pores in the coating were 175.0 μm, 169.3 μm and 172.5 μm, respectively, which were increased by 20.2%, 3.42% and 2.43% compared with 145.6 μm, 163.7 μm and 168.4 μm at 0 freeze–thaw cycles. When the polypropylene fiber content in TFC increased from 0%, 0.2% to 0.4% at 30 freeze–thaw cycles, the average diameters of the corresponding macroscopic pores in TFC were 185.3 μm, 180.6 μm, and 176.8 μm, respectively, which were increased by 27.27%, 10.32% and 4.99% compared with 0 freeze–thaw cycles. From Figure 6b, at 0 and 15 freeze–thaw cycles, the porosity of the coating with 0.2% polypropylene fiber was the smallest, but at 30 freeze–thaw cycles, the porosity was the largest. From Figure 8, the proportion of large pore size increased with the increase of freeze–thaw cycles. At 0 freeze–thaw cycles, the pore distribution of the D6 sample was 42.26% in the pore diameter range of 0.1–0.3 mm.

**Figure 9:**
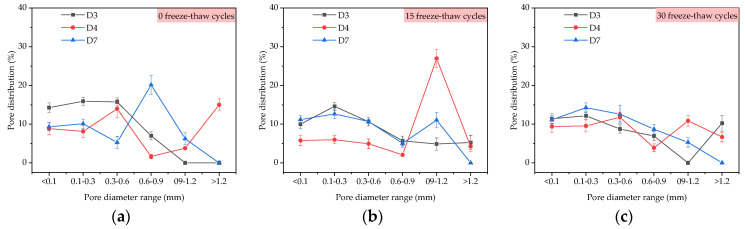
Pore distribution under the influence of air-entraining agent: (**a**) 0 freeze-thaw cycles; (**b**) 15 freeze-thaw cycles; (**c**) 30 freeze-thaw cycles.

By comparing D3, D4 and D7 samples in Figure 6a, it was shown that the average diameter and porosity of macroscopic pores increased with the increase of the content of the air-entraining agent, which was mainly due to the introduction of bubbles with the diameter of 25–250 μm by the air-entraining agent. When freeze–thaw cycles reached 15 times, the average diameters of the corresponding macropores were 175.2 μm, 169.3 μm and 174.0 μm, which were increased by 7.55%, 3.42% and 0%, respectively, compared with 162.9 μm, 163.7 μm and 174.5 μm under 0 freeze–thaw cycles. When freeze–thaw cycles reached 30 times, the average diameters of the corresponding macroscopic pores were 190.6 μm, 180.6 μm, and 180.9 μm, which were 17.00%, 10.32% and 3.67% higher than that under 0 freeze–thaw cycles, respectively. The air-entraining agent could still inhibit the deterioration of macropores, and it enhanced the freeze–thaw resistance of micropores of TFC. From Figure 6b, at 15 freeze–thaw cycles, the porosity of the coating with 0.015% air-entraining agent was the smallest, but it was only about 1% less than 0% and 0.030% content. From Figure 9, the pore distribution of the D7 sample with a pore diameter greater than 0.9 mm decreased significantly at 30 freeze–thaw cycles.

### 3.6. Mechanism Analysis

**Figure 10:**
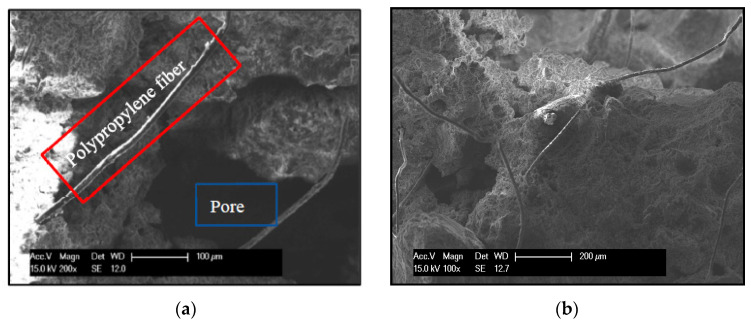
Environmental scanning electron microscope (ESEM) scanning electron microscope sampling: (**a**)enlargement of 200 times; (**b**)enlargement of 100 times.

The internal structure of the coating can be clearly seen in Figure 10. The addition of redispersible emulsion powder made the coating form a network structure, filled the internal pores and made it denser. Hence, from Figure 1a, the maximum bond strength was obtained when the content of redispersible emulsion powder was 4% under the same freeze–thaw cycles. At the same time, the mass-loss rate and damage degree of the coating with 2% redispersible emulsion powder was also reduced compared with that of 0%, according to Figure 2a and Figure 3a. Moreover, from Figure 4 and Figure 5 that the cumulative pore volume and pore diameter decreased with the increase of the content.

From Figure 10, the long fibrous material is polypropylene fiber, which connects the internal structure of the coating to form a whole to resist stress damage. Polypropylene fiber not only can share part of frost heaving stress but also has good resistance to damage caused by freeze–thaw cycle. Moreover, it has a bridge effect on cement-based coatings, which makes the internal microcracks of TFC difficult to form and improves the bond strength of TFC. Hence, from Figure 1b, the bond strength of 0.2% polypropylene fiber was close to that of 0.4% but greater than that of 0%.

The air-entraining agent introduced a large number of micropores, which can increase frost resistance. From Figure 10 that there were a large number of pores in the coating, but there were many small diameter pores. Air-entraining agent reduces the density of the coating, so the bond strength of 0.030% air-entraining agent was lower than that of 0.015% from Figure 1c. However, the damage degree and mass-loss rate decrease greatly under freeze–thaw cycles. The air-entraining agent could still inhibit the deterioration of macropores, and it could enhance the freeze–thaw resistance of micropores of TFC.

### 3.7. Bond Strength Model

After the parameter values of $p_{1}$ and $p_{2}$ were calculated by MATLAB (Equation (3)) according to the regression algorithm, the regression equations of Equations (5) and (6) could be obtained by lstopt software based on the universal global optimization (UGO).

(4) $$\sigma =p_{1}\ln\left(D\right)+p_{2}$$

(5) $$p_{1}=-0.0185-0.3969\times \alpha_{1}-1.8437\times \alpha_{2}+57.9833\times \alpha_{3}+3.1625\times \alpha_{1}{}^{2}+343.745\times \alpha_{2}{}^{2}-125111.1111\times \alpha_{3}{}^{2}$$

(6) $$p_{2}=0.0096+6.2645\times \alpha_{1}+29.02\times \alpha_{2}+445.6\times \alpha_{3}-101.3750\times \alpha_{1}{}^{2}-5550.0\times \alpha_{2}{}^{2}-1129777.7778\times \alpha_{3}{}^{2}$$

σ is the bond strength; D is the damage degree, taking the average value of $D_{L}$ and $D_{C}$; $p_{1}$ and $p_{2}$ are regression parameters; $\alpha_{1}$, $\alpha_{2}$ and $\alpha_{3}$ respectively represent thecontents of redispersible emulsion powder, polypropylene fiber and air entraining agent.

## 4. Conclusions

(1) With the increase of freeze–thaw cycle, the bond strength of the coatings with the same content of redispersible emulsion powder decreased. The bond strength increased with the increase of redispersible emulsion powder content under the same freeze–thaw cycles. The mass-loss rate and damage degree of the coating with 2% and 4% redispersible emulsion powder were also reduced compared with that of 0%;
(2) Polypropylene fibers not only can share part of frost heaving stress but also had good resistance to damage caused by freeze–thaw cycle. The bond strength of 0.2% polypropylene fiber was close to that of 0.4% but greater than that of 0%;
(3) The bond strength of 0.030% air-entraining agent was lower than that of 0.015% but greater than that of 0%. The damage degree and mass-loss rate decrease greatly under freeze–thaw cycles;
(4) In the freeze–thaw cycles, the most beneficial effect on the bond strength of coatings was redispersible emulsion powder, followed by air-entraining agent, and then polypropylene fiber;
(5) The mathematical model between bond strength and damage degree was established by Matlab.

Although there is a good correlation between bond strength and damage degree, further research is needed to verify and improve its reliability. In addition, a microscopic test was added to further study the mechanism of TFC.

## Figures and Tables

**Table 1:** Performance indexes of P.O.42.5R cement.

Apparent Density (kg/m^3^)	Specific Surface Area (m^2^/kg)	Loss on Ignition (%)	Initial/Final Setting Time/min	Flexural Strength (MPa)		Compressive Strength (MPa)	
				3d	28d	3d	28d
3050	360	1.06	125/185	5.7	8.4	27.5	45

**Table 2:** Composition of P.O.42.5R cement.

Components	Clinker	Gypsum Dihydrate	Fly Ash	Limestone	Slag
Content (%)	82.5	5.5	4.0	4.0	4.0

**Table 3:** Composition of high-alumina cement.

Components	SiO_2_	Al_2_O_3_	Fe_2_O_3_	R_2_O	Others
Content (%)	7.41	51.10	2.19	0.32	38.98

**Table 4:** Physical and chemical properties of redispersible emulsion powder.

Solid Content (%)	Specific Gravity (mL/g)	Ash Content (%)	Glass Transition Temperature (°C)	Particle Size (mm)	Viscosity (cps)
99 ± 1	7.41	10 ± 2	−4	0.115	10 s (1%)

**Table 5:** Main technical indexes of polypropylene fiber.

Item	Specifications	Item	Specifications
Fiber diameter	30 μm	Fiber length	6 mm
Tensile strength	≥350 MPa	Tensile break strength	>15%
Melting point	165–175 °C	Ignition point	590 °C
Density	0.91 kg/m^3^	Dispersion	Excellent
Acid and alkali resistance	Strong	Aging resistance	Strong
Electrical conductivity	Low	Security	Non-toxic

**Table 6:** Parameter levels.

Parameter		$\alpha_{1}$	$\alpha_{2}$	$\alpha_{3}$
Level	1	0%	0%	0%
	2	2%	0.2%	0.015%
	3	4%	0.4%	0.030%

**Table 7:** Test mix proportions of tunnel fireproof coatings (TFC) (%).

Sample	M1	M2	M3	M4	M5	M6	M7	M8	M9	M10	M11	M12	M13	M14	M15	M16
D1	35.10	5.10	1.22	0.51	15.41	12.96	7.76	2.55	3.27	9.90	1.02	2.04	2.96	0	0.2	0.015
D2	34.47	5.01	1.20	0.50	15.13	12.72	7.61	2.50	3.21	9.72	1.00	2.01	2.91	2	0	0.015
D3	34.40	5.00	1.20	0.50	15.10	12.70	7.60	2.50	3.20	9.70	1.00	2.00	2.90	2	0.2	0.030
D4	34.40	5.00	1.20	0.50	15.10	12.70	7.60	2.50	3.20	9.70	1.00	2.00	2.90	2	0.2	0.015
D5	33.69	4.90	1.18	0.49	14.79	12.44	7.44	2.45	3.13	9.50	0.98	1.96	2.84	4	0.2	0.015
D6	34.33	4.99	1.20	0.50	15.07	12.67	7.58	2.49	3.19	9.68	1.00	2.00	2.89	2	0.4	0.015
D7	34.40	5.00	1.20	0.50	15.10	12.70	7.60	2.50	3.20	9.70	1.00	2.00	2.90	2	0.2	0

Note: M1–M16 in the table correspond to P.O. 42.5, high-alumina cement, polyvinyl alcohol, polyvinyl alcohol silyl powder, expanded vermiculite, expanded perlite, sepiolite, hollow floating bead, Mg(OH)_2_, Al(OH)_2_, melamine, pentaerythritol, ammonium polyphosphate, redispersible emulsion powder, polypropylene fiber and air-entraining agent, respectively.

## Data Availability

Data sharing not applicable.
